# Dangerous Burning Oils

**Published:** 1874-10

**Authors:** 


Dangerous Burning Oils.—The Kentucky Legislature, at its last session, passed an act to protect the citizens of that commonwealth from explosive oils, which we hope will be carried out rigidly and imitated generally. * * *

An inspector, in the performance of his duties, shall use the standard instruments in use for that purpose, and shall test all oils as follows:

*First.* The water-cup should have sufficient water in it to rise two-thirds up the side of the oil-cup.

*Second.* Fill the oil-cup with oil to be tested to within one-eighth of an inch of the top.

*Third.* Suspend the thermometer so the bulb is just under the surface of the oil.

*Fourth.* Use an alcohol lamp to heat the water-bath, and before placing the light under the water-cup, test the oil in the oilcup by bringing a lighted match in contact with the surface of the oil. If it does not ignite, place the lamp under the water cup, and slowly heat the oil, not slower than one degree of the thermometer in a minute, nor faster than two degrees of the thermometer in a minute, moving a lighted match across the surface of the oil at each degree the thermometer rises, not more than three-eighths of an inch from the surface of the oil. If the oil should flash, that is, a little gas burn on the surface, and go out again, remove the lamp, and as soon as the thermometer ceases to rise, test the oil; and should it not ignite, replace the lamp, and test the oil each degree the thermometer rises until the oil ignites or permanently burns. As soon as the oil ignites or permanently burns, the degree indicated by the thermometer is the fire test of the oil. The flame moved across the surface of the oil should not exceed that of an ordinary match.

All oils and fluids specified in section one of this act that ignite or permanently burn at a temperature of one hundred and thirty degrees Fahrenheit and upward shall be approved by the inspector; and the barrels, casks, or packages containing the same shall be branded or marked by him with his name, official character, and the words "*standard oil;*" and all oils and fluids aforesaid, that ignite or permanently burn at a less temperature than one hundred and thirty degrees Fahrenheit shall be condemned by the inspector, and the barrels, casks or packages containing the same shall be branded or marked by him with his name, official character, and the words "unsafe for illuminating purposes."—*Medical and Surgical Reporter.*

By way of diversion, we present our readers with the following choice specimen of Homœopathic inductive reasoning.—Ed.

